# Novel roles for Arabidopsis dynamin-related proteins DRP1A and DRP2B in resistance against *Botrytis cinerea* fungal infection

**DOI:** 10.1080/15592324.2022.2129296

**Published:** 2022-10-06

**Authors:** Grant Mc Gowan, Gayani Ekanayake, Robert A. Ingle, Antje Heese

**Affiliations:** a Department of Molecular and Cell Biology, University of Cape Town, Rondebosch, South Africa; b University of Missouri-Columbia, Division of Biochemistry, Interdisciplinary Plant Group (IPG), Columbia, MO, USA

**Keywords:** CCV, dynamin, FLS2, flg22, plant immunity

## Abstract

Arabidopsis DYNAMIN-RELATED PROTEIN1A (*At*DRP1A) and *At*DRP2B are large GTPases that function together in endocytosis for effective cytokinesis, cell enlargement and development. A recent study shows that these DRPs contribute to ligand-induced endocytosis of the immune receptor FLAGELLIN SENSING2 (*At*FLS2) to modulate flg22-immune signaling, and they are required for immunity against *Pseudomonas syringae* pv. *tomato* bacteria. Here, we demonstrate that *atdrp1a* and *atdrp2b* single mutants showed increased susceptibility to *Botrytis cinerea* indicating that *At*DRP1A and *At*DRP2B are necessary for effective resistance against this necrotrophic fungus. Thus, we expanded our limited understanding of clathrin endocytic accessory proteins in immunity against plant pathogens.

## Main text

Endocytosis is the process by which eukaryotic cells take up extracellular components and plasma membrane (PM)-localized proteins to regulate cell surface-derived responses. During ligand-induced endocytosis, receptors are removed from the PM in response to specific ligands to desensitize cells to the stimulus and attenuate signaling.^1–3^ Plant studies have focused on clathrin-mediated endocytosis (CME), during which evolutionarily conserved and plant-specific clathrin core, adaptor and accessory proteins are recruited to the PM in a coordinated spatiotemporal manner.^1,3^

In eukaryotes, dynamins and DYNAMIN-RELATED PROTEINs (DRPs) are high-molecular weight GTPases functioning in the mechanochemical fission of organelles or membranes. *Arabidopsis thaliana* (Arabidopsis) DRPs are divided into six subfamilies with DRP1 and DRP2 family members being required for CME. Based on their domain structure, the plant-specific *At*DRP1 family contains five members (DRP1A-1E) whereas the evolutionary conserved *bone fide* dynamin family consists of the two *At*DRP2 members (*At*DRP2A-2B).^4,5^ *At*DRP1A and *At*DRP2B are the best studied DRPs contributing to constitutive CME of PM proteins and bulk PM for effective cytokinesis, cell enlargement and developmental processes.^3–5^ Consistent with their colocalization and biochemical interaction,^5^ we recently uncovered synergistic genetic interactions between *At*DRP1A and *At*DRP2B in plant growth and development as *atdrp1a atdrp2b* double mutants exhibit severely stunted roots and cotyledons, defective cell shape, cytokinesis and seedling lethality.^6^ In the absence of any stimulus, these double mutants also hyperaccumulate the Arabidopsis immune receptor FLAGELLIN SENSING2 (*At*FLS2) in the PM indicating combinatorial roles for *At*DRP1A and *At*DRP2B in governing PM abundance of *At*FLS2.^6^

Using single loss-of-function *atdrp* mutants, we have also shown that a) like *At*DRP2B,^7^ *At*DRP1A is required for effective defense signaling in response to flg22, a *Pseudomonas* flagellin peptide that is perceived by *At*FLS2 and induces host defense signaling; and b) *At*DRP1A plays a more prominent role than *At*DRP2B in flg22-induced endocytosis of FLS2.^6^ Consistent with ligand-induced endocytosis of FLS2 attenuating flg22-signaling,^8^ the delay in flg22-induced removal of FLS2 from the PM correlates with increased early flg22-signaling in *atdrp1a* and *atdrp2b* single mutants.^6,7^ *At*DRP1A and *At*DRP2B also contribute to effective immunity against bacterial pathogens because *atdrp1a* and *atdrp2b* single mutants display increased susceptibility to flagellated *Pseudomonas syringae* pv. *tomato* (*Pto*) DC3000 bacterial strains.^6,7^ The roles of DRP1A and DRP2B orthologues in defense signaling and host immunity extend to other pathogens; however, these DRPs have distinct contributions depending on the type of stimuli or pathogens.^9–12^ So far, however, it remains unknown whether *At*DRP1A and/or *At*DRP2B contribute to immunity against fungal infection.

**Figure 1.**
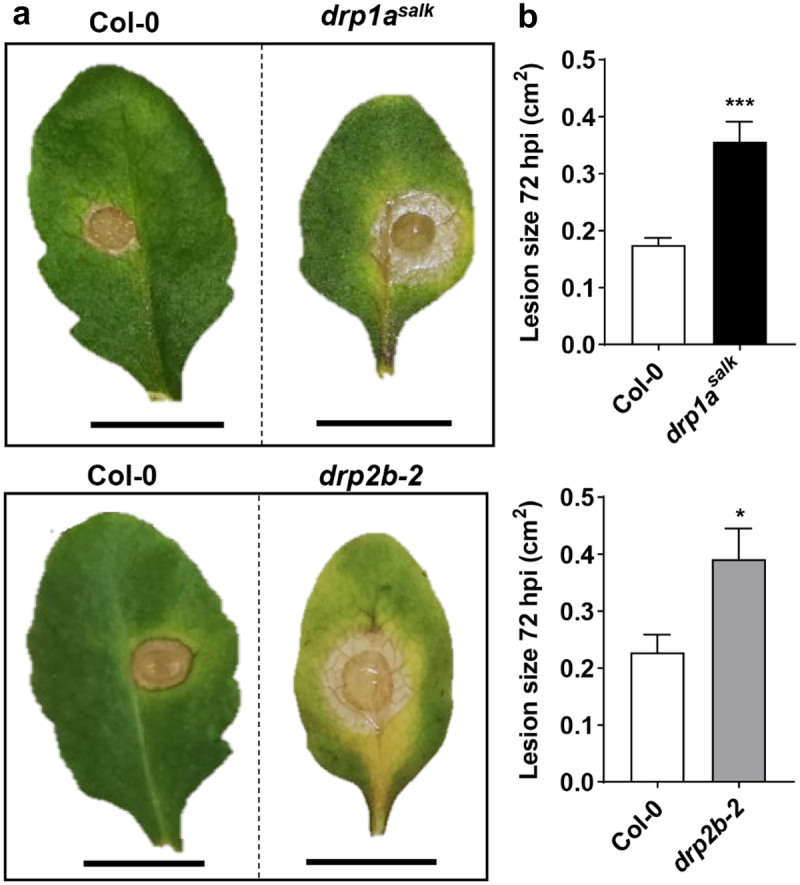
Single *atdrp1a* and *atdrp2b* mutants are more susceptible to the necrotrophic fungus *Botrytis cinerea*. Detached leaves from four-week-old Col-0 (wildtype), *atdrp1a^salk^* and *atdrp2b-2* single mutant plants were inoculated with *B. cinerea* spores for 72 h. (a) Representative leaves with lesions at 72 h post-infection (hpi). Stippled line indicates images taken from same experiment. Scale bar, 1 cm. (b) Lesion size (cm^2^) was measured at 72 hpi (n ≥ 15/ genotype), and values presented are means plus standard error of the mean (SEM). Using Student’s t-test, statistically significant differences were identified between Col-0 and *atdrp1a^salk^* (*P*= .0001) or *drp2b-2* (*P* = .0134). All experiments were performed five times with similar results.

To test this, we infected *atdrp1a* and *atdrp2b* single mutants with *Botrytis cinerea*, a highly destructive necrotrophic fungal pathogen with a broad host range including agricultural crops.^13^ We focused on altered susceptibility against this fungus in the single mutant plants because of severe seedling stunting and lethality of *drp1a drp2b* double mutants.^6^ Arabidopsis Col-0 (wild-type), *atdrp1a^salk^* (SALK_069077)^6,14^ and *atdrp2b-2* (SALK_134887)^7,15^ seeds were sown onto a 1:1 mixture of peat (Jiffy Products, Norway) and vermiculite. After stratification in the dark at 4°C for 3 days, plants were grown under 16 h light/8 h dark at 22°C and cool white fluorescent light of 80–100 µmol m^−2^ s^−1^. *Botrytis cinerea* pepper isolate was sub-cultured on apricot halves in the dark at 25°C two weeks prior to use of the spores. To determine the susceptibility of Col-0, *atdrp1a^salk^* and *atdrp2b-2* plants to *B. cinerea*, a maximum of two detached leaves from individual four-week-old plants were inoculated with 10 μL of half-strength grape juice containing 5 × 10^4^ spores mL^−1^ as previously described.^16^ Special care was taken to select leaves at a similar developmental stage and age, in that the youngest fully expanded leaves from each genotype were chosen for the fungal infection assays. Half-strength grape juice served as the mock infection control. Lesions were photographed 72 h post-inoculation (hpi) (Figure 1a). The size of each lesion was determined using IMAGEJ (http://rsbweb.nih.gov/ij/). Lesion areas were transformed by square rooting to meet assumptions of normality for parametric tests prior to statistical analysis using Student’s t-test (Figure 1b). As shown in Figure 1, *B. cinerea*-induced lesions were significantly larger for *atdrp1a^salk^*and *atdrp2b-2* single mutant leaves compared to Col-0 indicating that *atdrp1a^salk^* and *atdrp2b-2* were more susceptible to this necrotrophic fungus. We conclude that both *At*DRP1A and *At*DRP2B contribute positively to effective resistance against *B. cinerea*. Prior to our study, the only other Arabidopsis DRP1/2 member implicated in plant immunity against fungal infection is *At*DRP1E, originally identified as *enhanced disease resistance3* (*edr3*).^17^ In contrast to loss-of-function mutations in *atdrp1a* and *atdrp2b* resulting in increased susceptibility (this study), *edr3* mutant plants display increased resistance to *Erysiphe cichoracearum* and *B. cinerea*,^17^ likely due to a potential gain-of-function point mutation in the GTPase domain of *At*DRP1E because *atdrp1e* null mutant plants do not show altered resistance to these fungal pathogens.^17^

In conclusion, we have expanded our limited understanding of the CME accessory proteins *At*DRP1A and *At*DRP2B in plant immunity, providing evidence that *At*DRP1A and *At*DRP2B contributed to effective resistance against the necrotrophic fungus *B. cinerea*. The increased susceptibility in *atdrp1a* and *atdrp2b* single mutants may be caused by incorrect cell surface accumulation of cellular components that play critical roles during early stages of fungal infection. In future studies, it will be interesting to determine whether these Arabidopsis DRPs regulate the abundance of PM proteins involved in fungal perception and/or signaling.
